# Comparative proteomic analysis reveals novel insights into the interaction between rice and *Xanthomonas oryzae* pv. *oryzae*

**DOI:** 10.1186/s12870-020-02769-7

**Published:** 2020-12-14

**Authors:** Fan Zhang, Fan Zhang, Liyu Huang, Dan Zeng, Casiana Vera Cruz, Zhikang Li, Yongli Zhou

**Affiliations:** 1 Institute of Crop Sciences/National Key Facility for Crop Gene Resources and Genetic Improvement, Chinese Academy of Agricultural Sciences, 12 South Zhong-Guan-Cun Street, Beijing, 100081 China; 2 Graduate School of Chinese Academy of Agricultural Sciences, 12 Zhong-Guan-Cun Street, Beijing, 100081 China; 3 School of Agriculture, Yunnan University, Kunming, 650091 China; 4 International Rice Research Institute, DAPO Box 7777, Metro Manila, The Philippines

**Keywords:** Rice, *Xanthomonas oryzae* pv. *oryzae*, Compatible interaction, Incompatible interaction, Quantitative proteome

## Abstract

**Background:**

Bacterial blight, which is caused by *Xanthomonas oryzae* pv. *oryzae* (*Xoo*), is a devastating rice disease worldwide. Rice introgression line H471, derived from the recurrent parent Huang-Hua-Zhan (HHZ) and the donor parent PSBRC28, exhibits broad-spectrum resistance to *Xoo*, including to the highly virulent *Xoo* strain PXO99^A^, whereas its parents are susceptible to PXO99^A^. To characterize the responses to *Xoo*, we compared the proteome profiles of the host and pathogen in the incompatible interaction (H471 inoculated with PXO99^A^) and the compatible interaction (HHZ inoculated with PXO99^A^).

**Results:**

In this study, a total of 374 rice differentially abundant proteins (DAPs) and 117 *Xoo* DAPs were detected in the comparison between H471 + PXO99^A^ and HHZ + PXO99^A^. Most of the *Xoo* DAPs related to pathogen virulence, including the outer member proteins, type III secretion system proteins, TonB-dependent receptors, and transcription activator-like effectors, were less abundant in the incompatible interaction than in the compatible interaction. The rice DAPs were mainly involved in secondary metabolic processes, including phenylalanine metabolism and the biosynthesis of flavonoids and phenylpropanoids. Additionally, some DAPs involved in the phenolic phytoalexin and salicylic acid (SA) biosynthetic pathways accumulated much more in H471 than in HHZ after the inoculation with PXO99^A^, suggesting that phytoalexin and SA productions were induced faster in H471 than in HHZ. Further analyses revealed that the SA content increased much more rapidly in H471 than in HHZ after the inoculation, suggesting that the SA signaling pathway was activated faster in the incompatible interaction than in the compatible interaction.

**Conclusions:**

Overall, our results indicate that during an incompatible interaction between H471 and PXO99^A^, rice plants prevent pathogen invasion and also initiate multi-component defense responses that inhibit disease development.

## Background

Bacterial blight (BB) caused by the Gram-negative bacterium *Xanthomonas oryzae* pv. *oryzae* (*Xoo*) is one of the most widely distributed and devasting diseases of rice (*Oryza sativa* L.), leading to substantial yield losses [1]. Developing and deploying broad-spectrum resistant varieties is the most economically viable BB management strategy. Therefore, further characterizing rice-*Xoo* interactions will provide insights useful for developing and breeding resistant cultivars.

Several types of *Xoo* virulence factors have been identified, including proteins associated with exopolysaccharide (EPS) production and motility, outer membrane (OM) proteins and TonB-dependent receptors, hypersensitive response and pathogenicity (Hrp) proteins, and type III (T3) effectors [2].

Plants have developed rapid and sophisticated mechanisms that confer resistance to invading pathogens. The cell surface immune receptors (CSIRs) and intracellular immune receptors (IIRs) directly or indirectly recognize apoplastic or cytoplasmic “invasion molecules” to induce weak or strong immune responses [3]. Activated defense responses consist of callose deposition in cell walls, oxidative burst, and accumulation of phytohormones and antimicrobial chemicals (e.g., secondary metabolites and phytoalexins) [4]. In response to pathogen infections, rice produces many phytoalexins, including diterpenoid compounds and the flavonoid sakuranetin [5]. A new class of phenolic phytoalexins with antimicrobial activities against bacterial and fungal pathogens in rice comprises stress-induced phenylamides, which rely on the shikimate pathway for aromatic L-amino acids and the phenylpropanoid pathway for the phenolic acid moieties in phenylamides and sakuranetin [6]. Salicylic acid (SA) is an important signaling hormone in plant defense responses, but it is also a phenolic compound synthesized by plants in response to diverse pathogens [7]. The triggered immunity involves considerable increases in the endogenous levels of SA and its conjugates prior to the induction of pathogenesis-related (PR) proteins and the onset of local and systemic acquired resistance [8]. Additionally, SA biosynthesis in higher plants occurs through the shikimate phenylpropanoid pathway, and may involve two distinct routes, the isochorismate (IC) pathway and the phenylalanine ammonia-lyase (PAL) pathway [9]. Many investigations proved that the bulk of SA is produced from IC via two reactions catalyzed by isochorismate synthase (ICS) and isochorismate pyruvate lyase (IPL) [10–12].

To date, 43 major resistance (*R*) genes conferring resistance to BB have been identified in rice [13–16]. Although there has been considerable research aimed at characterizing the molecular mechanism mediated by *R* genes, the interactions between rice and *Xoo* at the genome level remain unclear. The rapid development of proteomics technology has enabled researchers to comprehensively explore the changes to protein abundances during physiological and biochemical processes. Over the last decade, researchers have applied diverse proteomic experimental techniques to investigate rice resistance to *Xoo*. For example, Mahmood [17] used two-dimensional polyacrylamide gel electrophoresis (2-DE) to separate rice cytosolic and membrane proteins extracted from plants inoculated with compatible or incompatible *Xoo* races. Chen [18] combined 2-DE and tandem mass spectrometry (MS/MS) analyses to identify plasma membrane proteins involved in the Xa21 signaling pathway. Wang [19] used 2-DE coupled with matrix-assisted laser desorption/ionization time-of-flight mass spectrometry (MALDI-TOF-MS) to identify proteins involved in defense responses to *Xoo*.

We previously identified the rice introgression line (IL) H471, which was derived from a hybridization between the susceptible recurrent parent Huang-Hua-Zhan (HHZ) and the donor parent PSBRC28. Line H471 reportedly undergoes a broad-spectrum hypersensitive response (HR) to BB mediated by a new rice resistance gene, *Xa39* [16]. In the present study, we conducted a differential proteome analysis of rice lines (H471 and its recurrent parent HHZ) and the strongly virulent *Xoo* strain PXO99^A^ using tandem mass tag (TMT) technology coupled with liquid chromatography-quadrupole mass spectrometry (LC-MS/MS) [20]. Our results provide novel information that clarifies the incompatible interactions between *Xoo* and rice that lead to HR.

## Results

### Reactions of H471 and HHZ in response to PXO99^A^

**Fig. 1:**
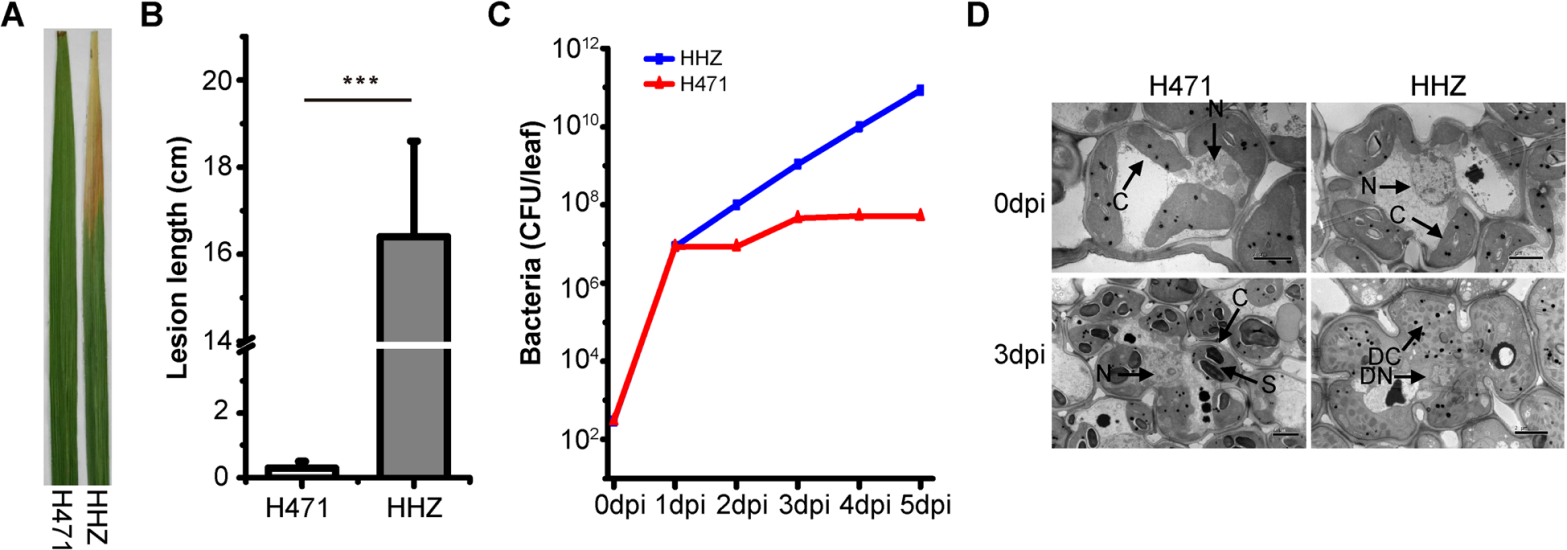
Phenotypic reactions of rice introgression line H471 and its recurrent parent Huang-Hua-Zhan (HHZ) to an infection by *Xanthomonas oryzae* pv. *oryzae* strain PXO99^A^. **a** Phenotypes of HHZ and H471 plants infected with *Xoo* strain PXO99^A^. Photographs were taken 14 days after inoculation. **b** Lesion lengths on H471 and HHZ plants infected with PXO99^A^ at 14 dpi. Data are presented as the mean of nine independent plants for each line; vertical bars indicate the standard deviation. *** significant differences between HHZ and H471 at *p* < 0.001 based on Student’s *t*-test. **c** Growth curves of PXO99^A^ in H471 and HHZ. CFU indicates colony-forming units. **d** Transmission electron microscopy analysis of H471 and HHZ mesophyll cells after the inoculation with PXO99^A^. N, nucleus; C, chloroplast; S, starch; DC, damaged chloroplast; DN, damaged nucleus

Rice introgression line (IL) H471 and its recurrent parent HHZ were evaluated following an inoculation with *Xoo* PXO99^A^ at the tillering stage. Distinct brown edges along the clipped sites of H471 were visible at 3 days post-inoculation (dpi), and the brown necrosis at the infection site became more apparent at 5 dpi. These observations indicated the IL exhibited a typical HR to *Xoo*. In contrast, on the infected HHZ leaves, chlorosis was visible at 3 dpi, and water-soaked lesions rapidly spread along the clipped sites at 5 dpi (Fig. 1a). The lesion lengths of H471 and HHZ at 14 dpi were 0.3 ± 0.2 cm and 16.4 ± 2.2 cm, respectively (Fig. 1b). Moreover, PXO99^A^ growth in H471 was markedly lower than that in HHZ as follows: more than 10-fold lower at 2 dpi, more than 20-fold lower at 3 dpi, about 190-fold lower at 4 dpi, and more than 1600-fold lower at 5 dpi (Fig. 1c). We also examined the ultrastructural changes in H471 and HHZ leaf cells 3 days after an inoculation with PXO99^A^ via transmission electron microscopy (TEM). Before the inoculation, there were no significant structural differences between the mesophyll cells of H471 and HHZ. However, at 3 dpi, the HHZ cell membranes were slightly damaged, and the outlines of the chloroplasts and mitochondria were unclear. In contrast, clear outlines of the whole cell and organelles were visible for H471, and many starch grains were detected (Fig. 1d). These results indicated that H471 is highly resistant to PXO99^A^ (incompatible interaction between H471 and PXO99^A^), whereas HHZ is highly susceptible (compatible interaction between HHZ and PXO99^A^).

### Protein identification and quantification

The molecular weight range was greater for the identified rice proteins (4000–469,100 Da) than for the identified *Xoo* proteins (5300–195,500 Da) (Additional files 1 and 2). The isoelectric points were 3.5–12.5 and 4.2–12.4 for most rice proteins and *Xoo* proteins, respectively (Additional files 1 and 2). To improve analytical precision, all quantitative data for peptides identified in multiple fractions were used to quantify proteins. A total of 1,289,153 MS/MS spectra were obtained and were approximately matched to 196,745 known peptide sequences (Additional file 3). Overall, we identified and quantified 46,076 peptides associated with 8120 different proteins (7784 rice proteins and 336 *Xoo* proteins) in the mixed proteomics samples (H471 + PXO99^A^ and HHZ + PXO99^A^) at 2 and 3 dpi (Additional files 1, 2, 3 and 4). A clustering analysis involving all identified proteins revealed that samples from the same genetic background clustered in the same groups (Additional file 5).

### Differentially abundant proteins between incompatible and compatible interactions

**Fig. 2:**
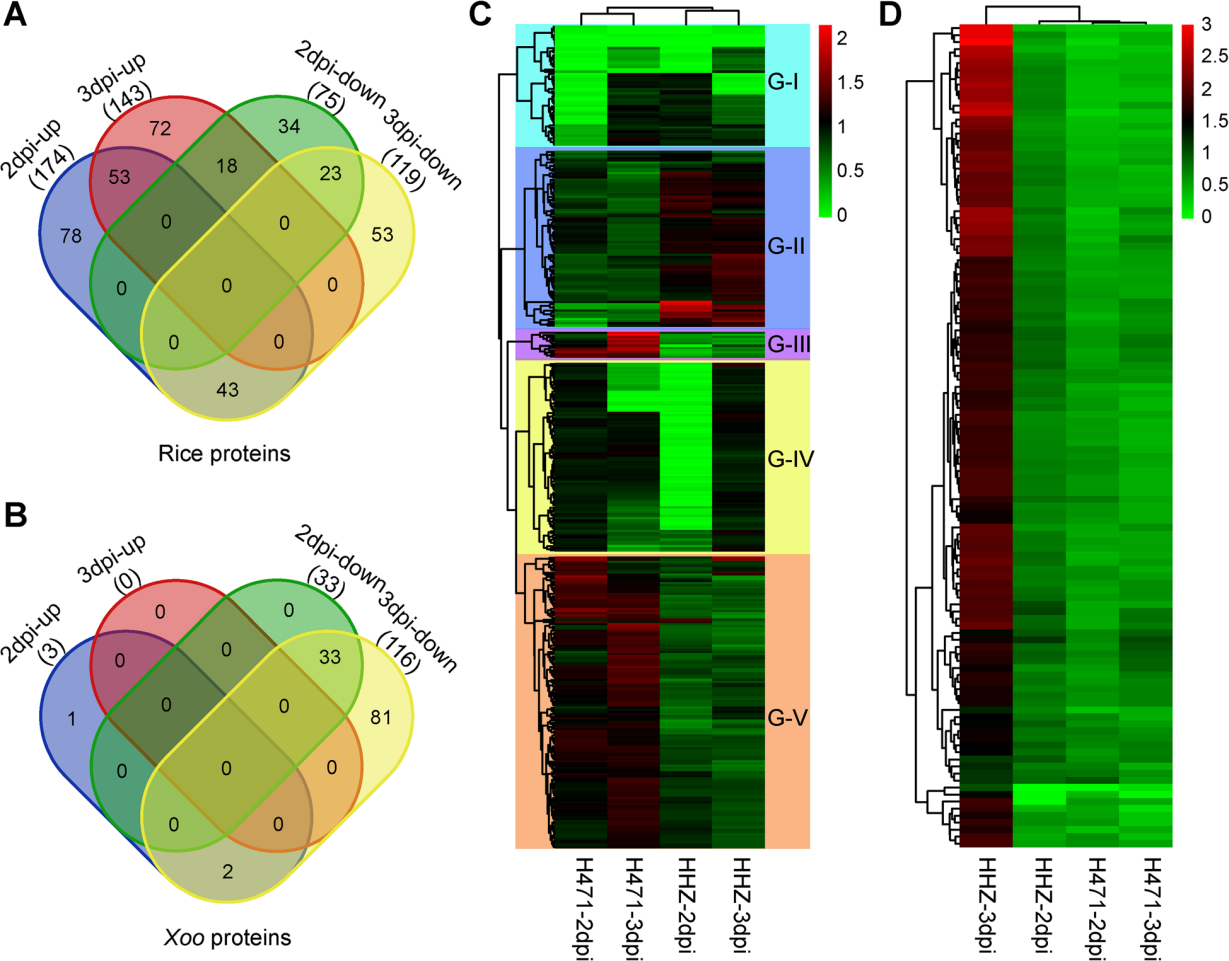
Differentially abundant proteins (DAPs) identified by pairwise comparisons between H471 and HHZ at two time-points after the inoculation with PXO99^A^. Venn diagrams of rice DAPs (**a**) and *Xanthomonas oryzae* pv. *oryzae* (*Xoo*) DAPs (**b**) between H471 and HHZ at two time-points. The individual and overlapping areas in the Venn diagrams represent the number of specific and common DAPs. Heat map of rice DAPs (**c**) and *Xoo* DAPs (**d**) between H471 and HHZ at two time-points after the inoculation with PXO99^A^. Each cell reflects the mean value of the normalized accumulation of one DAP in three biological replicates per sample. Rice DAPs are divided into five main clusters, namely G-I to G-V

**Fig. 3:**
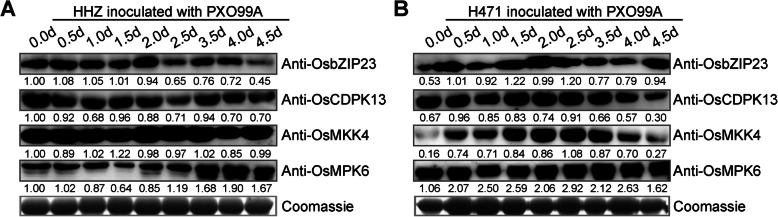
Abundances of OsbZIP23, OsCDPK13, OsMKK4, and OsMPK6 in HHZ and H471 after the inoculation with PXO99^A^. (**a**) and (**b**) represent the protein abundances in HHZ and H471, respectively, as determined in a western blot analysis. The relative OsbZIP23, OsCDPK13, OsMKK4, and OsMPK6 abundances were analyzed with Image J. Full-length blot images were presented in Additional file 12. The experiments were repeated three times, with similar results

Only unique peptides were considered for quantifying proteins, resulting in the identification of 374 rice DAPs and 117 *Xoo* DAPs. Additionally, 264 rice DAPs and 3 *Xoo* DAPs accumulated more in H471 + PXO99^A^ than in HHZ + PXO99^A^, with a threshold fold-change > 1.5 and *p* < 0.05 in *t*-tests, whereas 171 rice DAPs and 116 *Xoo* DAPs accumulated less in H471 + PXO99^A^ than in HHZ + PXO99^A^, with a threshold fold-change < 0.67 and *p* < 0.05 in *t*-tests (Fig. 2a, b, Additional files 6 and 7). Furthermore, 249 rice DAPs and 36 *Xoo* DAPs were detected between H471 + PXO99^A^ and HHZ + PXO99^A^ at 2 dpi, whereas 262 rice DAPs and 116 *Xoo* DAPs were detected between H471 + PXO99^A^ and HHZ + PXO99^A^ at 3 dpi. A total of 137 rice DAPs and 35 *Xoo* DAPs were common between the two time-points (Fig. 2a and b). The hierarchical clustering of all rice DAPs indicated that rice DAPs in HHZ at 2 and 3 dpi were clustered in one subgroup, whereas those in H471 at 2 and 3 dpi were in another subgroup (Fig. 2c). The clustering of all *Xoo* DAPs suggested that *Xoo* DAPs in H471 at 2 and 3 dpi were clustered in one subgroup, whereas DAPs in HHZ at 2 dpi were grouped with those in H471 (Fig. 2d, Additional file 6). On the basis of protein abundances, the 374 rice DAPs (Additional file 7) were classified into five groups (G-I to V). The proteins in G-III and G-V were more abundant in H471 than in HHZ, whereas the opposite trend was observed for the DAPs in G-II (Fig. 2c). We also extracted the total proteins of HHZ and H471 at 0, 0.5, 1, 1.5, 2, 2.5, 3.5, 4, and 4.5 dpi, and used specific antibodies against four of the obtained DAPs to validate their abundances at different time-points. The western blot results were highly consistent with the proteomic experiments (Fig. 3). However, the data obtained in the present proteomic study was poorly correlated with the results of our previous transcriptomic analyses, implying there are specific mechanisms for maintaining proper levels of transcripts and proteins [21].

The application of the KOBAS platform to identify enriched GO terms and pathways revealed that *Xoo* DAPs in H471 were significantly enriched with 14 GO terms, and ribosomal proteins (22.5%) formed the largest set of modulated proteins (Additional file 8). The functional classification of these proteins indicated that most were involved in structural molecule activity and RNA binding. Most of the proteins assigned to the biological process category were involved in translation, protein folding, and generation of precursor metabolites and energy (Additional file 8). The significantly enriched KEGG pathways in *Xoo* were those related to the ribosome (xop03010, *p* = 1.4E**−** 14), RNA degradation (xop03018, *p* = 1.3E**−** 3), methane metabolism (xop00680, *p* = 9.51E**−** 03), gluconeogenesis (xop00010, *p* = 1.71E**−** 02), carbon metabolism (xop01200, *p* = 2.75E**−** 02), and the TCA cycle (xop00020, *p* = 2.89E**−** 02) (Additional file 9). Regarding the rice DAPs, the three enriched GO terms were secondary metabolic processes, endopeptidase inhibitor activity, and peptidase inhibitor activity, and the five enriched KEGG pathways were biosynthesis of secondary metabolites (osa01110, *p* = 6.74E**−** 03), phenylalanine metabolism (osa00360, *p* = 1.23E**−** 02), flavonoid biosynthesis (osa00941, *p* = 3.52E**−** 02), phenylpropanoid biosynthesis (osa00940, *p* = 4.31E**−** 02), and vitamin B6 metabolism (osa00750, *p* = 4.55E**−** 02) (Additional files 8 and 9).

### Differentially abundant *Xoo* proteins between the incompatible and compatible interactions

**Fig. 4:**
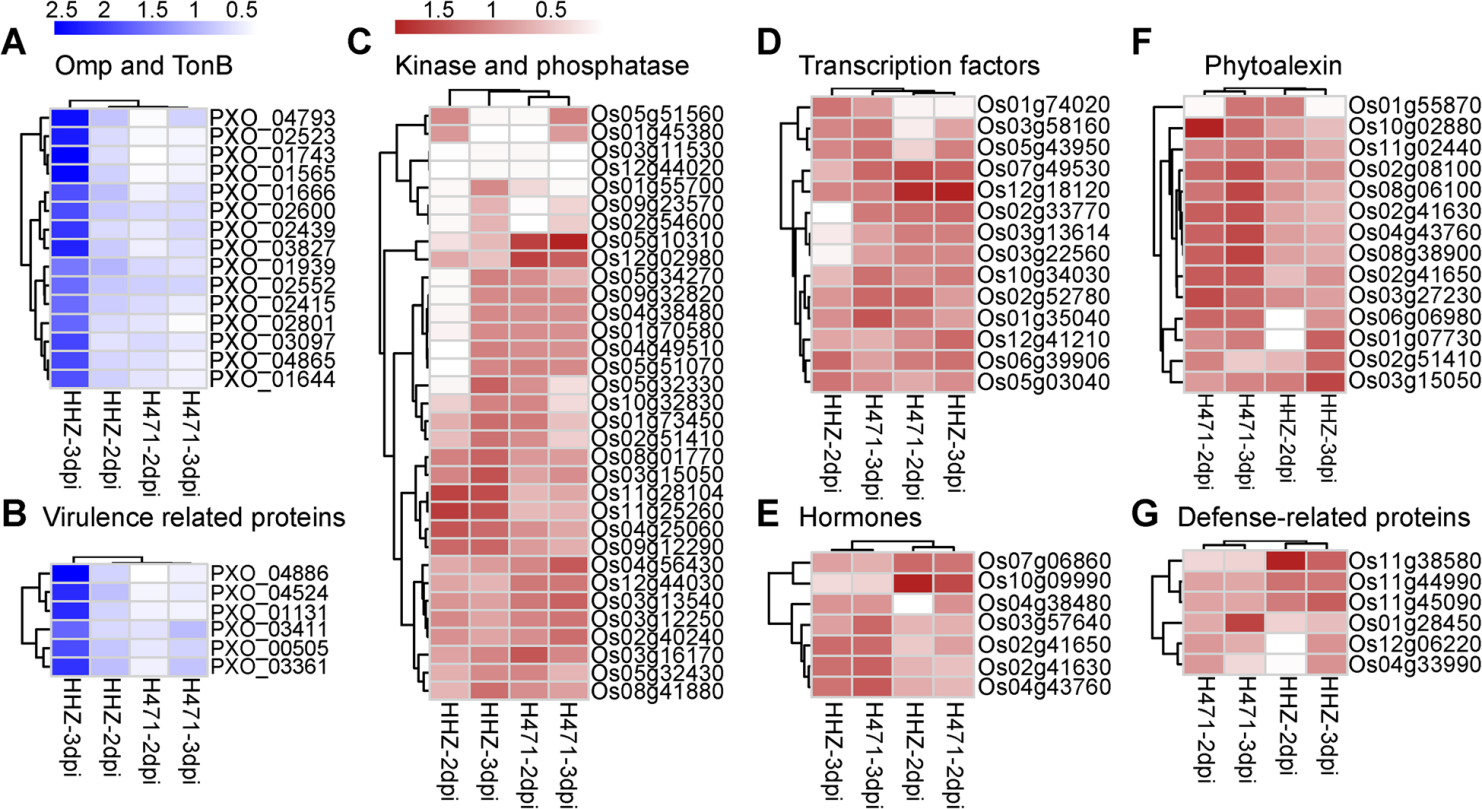
Patterns of the *Xanthomonas oryzae* pv. *oryzae* (*Xoo*) and rice differentially abundant proteins assigned to six main categories putatively related to incompatible and compatible interactions. **a** Outer membrane proteins (Omp) and TonB-dependent receptors (TonB). **b** Virulence-related proteins in *Xoo*. **c** Kinases and phosphatases. **d** Transcription factors. **e** Phytohormone-related proteins. **f** Phytoalexin-related proteins. **g** Defense response proteins. Red and blue represent *Xoo* and rice proteins, respectively

On the basis of protein functions, the detected *Xoo* DAPs were grouped into the following categories: type III secretion system (T3SS), transcription activator-like (TAL) effector, nutrient uptake, and uncharacterized protein. In this study, the abundances of six outer membrane proteins (Omps) and nine TonB-dependent receptor-related proteins were significantly different between H471 and HHZ mainly at 3 dpi (12/15) (Fig. 4a). Similarly, one T3SS-related protein (HrpE, PXO_03411), one TAL effector (talC3b, PXO_00505), and a VirK protein (PXO_03361) accumulated much more in HHZ-3d than in HHZ-2d and H471-3d. In this study, the VirK protein (PXO_03361) accumulated less in H471 than in HHZ at 2 and 3 dpi. Additionally, elongation factors (PXO_04524 and PXO_01131) were also less abundant in H471 than in HHZ at 3 dpi (Fig. 4b). Increases in the contents of these *Xoo* DAPs were more pronounced in HHZ at 3 dpi than at 2 dpi, and the fold-changes of these protein abundances were significantly greater in HHZ-3d vs HHZ-2d than in H471-3d vs H471-2d.

### Differentially abundant rice proteins between the incompatible and compatible interactions

In addition to the unknown proteins, the rice DAPs identified in the comparison between H471 and HHZ were grouped into the following five categories: signal transduction, transcription, phytohormone, phytoalexin, and defense response. Hierarchical clustering provided an overview of the differential abundance patterns of the protein types (Fig. 4c-g).

Protein kinases and phosphatases are key co-regulators of protein phosphorylation, and are particularly prominent in signal transduction pathways. In this study, 21 differentially abundant protein kinases and 12 differentially abundant protein phosphatases were identified between the incompatible and compatible interactions (Fig. 4c). Calcium-dependent protein kinases (CDPKs) function as Ca^2+^ sensors and effectors that relay specific Ca^2+^ signatures to downstream components via CDPK-dependent protein phosphorylation. In this study, the accumulation of OsCDPK7/OsCDPK13 (LOC_Os04g49510) was more than 200 times greater in H471 than in HHZ at 2 dpi (Fig. 4c, Additional file 7). Likewise, a western blot assay indicated that OsCDPK13 accumulated more in H471 than in HHZ at 2.5 dpi (Fig. 3). The abundances of two CDPK-related kinases (CRKs), LOC_Os04g25060/LOC_Os04g25650 and OsCRK5 (LOC_Os04g56430), were also significantly different between H471 and HHZ. Additionally, the accumulation of a somatic embryogenesis receptor kinase (SERK), OsSERK2 (LOC_Os04g38480), was approximately 10-fold greater in H471 than in HHZ at 2 dpi (Fig. 4c, Additional file 7). The mitogen-activated protein kinase (MAPK) cascades comprise three protein kinase components, MAPK, MAPK kinase (MAPKK), and MAPKK kinase (MAPKKK). The OsMKK4 (LOC_Os02g54600) abundance was lower in H471 than in HHZ at 2 dpi (Fig. 4c, Additional file 7). As indicated in Fig. 3, OsMKK4 accumulated less in H471 than in HHZ. Moreover, it was maintained at a relatively high abundance in HHZ, whereas in H471, it accumulated at 0.5 dpi, peaked at 2 dpi, and then decreased at 4 dpi. In the proteomic assays, there was no obvious difference in OsMPK6 accumulation between HHZ and H471 at 2 and 3 dpi. However, the western blot assay involving the OsMPK6-specific antibody suggested that the accumulation of OsMPK6 significantly increased after HHZ and H471 were inoculated with PXO99^A^, and this protein accumulated much more in H471 than in HHZ from 0.5 to 4.0 dpi (Fig. 3). Additionally, 14 protein phosphatases differentially accumulated in H471 and HHZ, including PP2C30 (LOC_Os03g16170) and two Ser/Thr protein phosphatases (LOC_Os03g13540 and LOC_Os12g44020).

Transcription factors (TF) play key roles in the large-scale transcriptional reprogramming of plants in response to pathogens. A comparison with HHZ revealed 14 DAPs encoding TFs in H471 infected by *Xoo* (Fig. 4d), including bZIP, MYB, zinc finger, AP2, homeobox, and HSF family members. Seven and five TFs were more and less abundant, respectively, in H471 than in HHZ at 2 or 3 dpi. The MYB family protein LOC_Os01g74020 accumulated significantly less in H471 than in HHZ at 2 dpi, but more in H471 than in HHZ at 3 dpi. A zinc finger family protein (LOC_Os12g18120) accumulated more and less in H471 than in HHZ at 2 and 3 dpi, respectively (Fig. 4d, Additional file 7). Additionally, two bZIP TFs, OsbZIP23 (LOC_Os02g52780) and LOC_Os03g13614, accumulated much more in H471 than in HHZ at 2 and 3 dpi, respectively (Fig. 4d, Additional file 7). The western blot assay result further confirmed that OsbZIP23 was more abundant in H471 than in HHZ at 2, 2.5, and 3 dpi (Fig. 3). Heat stress TFs (Hsfs) also regulate gene expression in response to environmental stress. In this study, OsHsfA2e (LOC_Os03g58160) and OsVOZ2 (LOC_Os05g43950) accumulated less in H471 than in HHZ at 2 dpi (Fig. 4d, Additional file 7).

Phytohormones are signaling molecules that circulate throughout plants and stimulate responses to various environmental stresses. The abundances of two gibberellin receptors (LOC_Os07g06860 and LOC_Os03g57640) differed between H471 and HHZ. Specifically, LOC_Os07g06860 was less abundant in H471 than in HHZ at both 2 and 3 dpi, whereas LOC_Os03g57640 was more abundant in H471 than in HHZ at 3 dpi. Cytokinin-O-glucosyltransferase 3 (LOC_Os10g09990) was less abundant in H471 than in HHZ at both time-points (Fig. 4e). Notably, the following three PALs accumulated much more in H471 than in HHZ: LOC_Os02g41630 and LOC_Os04g43760 at 2 and 3 dpi, and LOC_Os02g41650 at 2 dpi (Fig. 4e).

Phytoalexin accumulation at an infection site is frequently associated with relatively broad-spectrum antimicrobial activity. Additionally, PAL is a key enzyme in the phenylpropanoid pathway contributing to phytoalexin synthesis, and it is also important for SA synthesis. We observed that several proteins involved in the phenylpropanoid pathway, including Os4CL3 (LOC_Os02g08100), two O-methyltransferases (OMTs) (LOC_Os10g02880 and LOC_Os08g06100), and two caffeoyl-CoA O-methyltransferases (COMTs) (LOC_Os06g06980 and LOC_Os08g38900), were considerably more abundant in H471 than in HHZ at one or both time-points after inoculation (Fig. 4f). Moreover, two DAPs associated with the shikimate pathway were identified. The accumulation of chorismate mutase (LOC_Os01g55870) was lower in H471 than in HHZ at 2 dpi, but higher at 3 dpi. In contrast, shikimate kinase (LOC_Os02g51410) accumulated much more in H471 than in HHZ at 2 dpi, but less at 3 dpi. Chalcone-flavonone isomerase (LOC_Os11g02440), which is related to sakuranetin biosynthesis, also accumulated more in H471 than in HHZ at 3 dpi (Fig. 4f). Furthermore, three proteins (LOC_Os03g15050, LOC_Os01g07730, and LOC_Os03g27230) involved in phosphoenol pyruvate biosynthesis accumulated differently in H471 and HHZ. The abundance of LOC_Os03g15050 was lower in H471 than in HHZ at 3 dpi, whereas the abundances of LOC_Os01g07730 and LOC_Os03g27230 were higher in H471 than in HHZ at 2 and 3 dpi. (Fig. 4f).

Three and two defense-related proteins were identified as less and more abundant, respectively, in H471 than in HHZ plants inoculated with *Xoo*. Two NB-ARC domain-containing protein family members (LOC_Os11g44990 and LOC_Os11g45090) and an NBS-LRR disease resistance protein (LOC_Os11g38580) accumulated less in H471 than in HHZ. Both OsPR1b (LOC_Os01g28450) and a harpin-induced protein (LOC_Os12g06220) accumulated much more in H471 than in HHZ. Notably, the accumulation of LOC_Os12g06220 was more than 160-times higher in H471 than in HHZ at 2 dpi (Fig. 4g, Additional file 7). Additionally, a harpin-induced protein (LOC_Os04g33990) accumulated more and less at 2 and 3 dpi, respectively, in H471 than in HHZ (Fig. 4g).

### Abundances of proteins associated with SA signaling during interactions between rice and *Xoo*

**Fig. 5:**
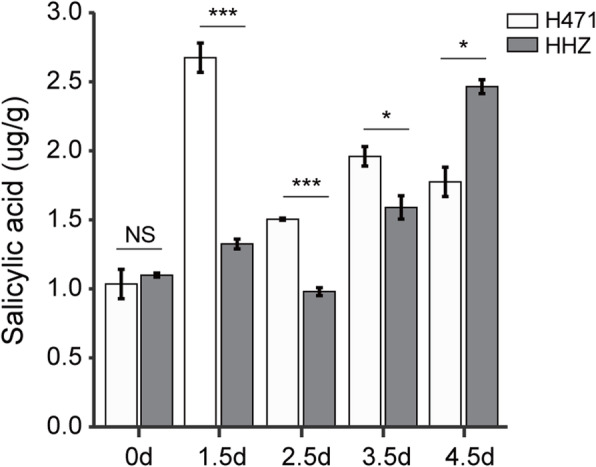
Free salicylic acid contents of HHZ and H471 plants inoculated with PXO99^A^. Data are presented as the mean (two replicates) ± standard deviation. NS *p* > 0.05, * *p* < 0.05, and *** *p* < 0.001

To further investigate whether SA contributes to the disease resistance of H471, we analyzed the abundances of the SA-inducible OsPR1a and OsPR1b. Western blot assays indicated that in plants inoculated with PXO99^A^, OsPR1a levels increased at 1 and 0.5 dpi in HHZ and H471, respectively, and were much higher in H471 than in HHZ from 2.5 to 4 dpi (Additional file 10 a). Because the accumulation of OsPR1b was too low to detect, we were unable to quantify its abundance in HHZ, and only unclear bands were detected at 2.5 and 4.5 dpi for H471 (Additional file 10 b). After the inoculation with PXO99^A^, the SA content in H471 increased rapidly and was double that in HHZ at 1.5 dpi. The SA content was much higher in H471 than in HHZ at 2.5 and 3.5 dpi, and it decreased in HHZ at 4.5 dpi (Fig. 5). These results suggested that the SA signaling pathway was activated in response to the *Xoo* infection in H471.

## Discussion

Proteomic strategies have recently been successfully applied to analyze plant disease resistance. A number of proteins associated with BB resistance responses in rice have been identified. However, little information is available on the global changes in pathogen proteins during plant-pathogen interactions. In this study, the DAPs of the plant host and pathogen were examined simultaneously. Specifically, quantitative proteomic technology was used to compare incompatible (H471 and PXO99^A^) and compatible (HHZ and PXO99^A^) interactions. The differences in the proteome profiles of rice and *Xoo* between the incompatible and compatible interactions revealed interesting molecular mechanisms underlying the broad-spectrum resistance of rice to BB.

### The abundances of proteins related to *Xoo* invasion and growth in rice were lower during the incompatible interaction than during the compatible interaction

Entry into host cells is a crucial step in bacterial infections. Bacterial pathogens use diverse virulence-related factors, including the T3SS, for host cell binding, the triggering of membrane ruffles, and cooperative invasions. The Omps of Gram-negative bacteria are essential for bacterial survival during eukaryotic cell invasions [22, 23]. The OmpA protein is required for bacterial infection/colonization and cellular adhesion/development in host cells [24]. Bacterial TonB-dependent receptors, which are involved in the uptake of nutrients from the surrounding environment, and extracellular enzymes are also important for the interactions between *Xoo* and rice [19]. An elongation factor is essential for viability and is required for protein synthesis. In this study, DAPs related to six Omps, nine TonB-dependent receptors, and two elongation factors were detected, and all of them accumulated less in H471 than in HHZ. These results suggested that PXO99^A^ invasion and growth were significantly more inhibited in the incompatible interaction than in the compatible interaction. The dramatically decreased *Xoo* population in H471 was consistent with the differential protein abundances.

The T3SS, encoded by *hrp* genes, plays an important role in the interaction between *Xoo* and rice because it injects T3 effectors into plant cells [25]. The *Xoo* T3 effectors include two collections of proteins, namely TAL effectors and *Xanthomonas* outer proteins (Xop). The TAL effectors can recognize and bind to specific DNA sequences within the promoters of corresponding host genes, resulting in the transcriptional activation of the genes mediating disease susceptibility or resistance [26]. In the current study, HrpE (PXO_03411) accumulated less in H471 than in HHZ. Notably, the T3 effectors talC3b (PXO_00505) and OsVOZ2 (LOC_Os05g43950) also accumulated less in H471 than in HHZ. A previous study proved that interactions between XopN_KXO85_ and OsVOZ2 increase rice susceptibility to *Xoo* [27]. However, in the present study, we were unable to identify the target gene of talC3b based on the list of rice DAPs. Additionally, the PXO99^A^ XopN protein abundance was similar in H471 and HHZ at the two examined time-points. This might have been due to a shift in the timing of gene expression as the host-pathogen interaction developed. We speculated that the effectors talC3b and XopN may serve as signaling molecules that modulate the host response to infection when they are released in plant tissues, wherein they activate their target genes whose protein products contribute to the susceptibility of the host plants in a compatible interaction. In contrast, these proteins do not mediate a susceptible response in incompatible interactions because of their lower abundances. Clarifying the functions of the two effectors and their host targets will be a key step for further characterizing the interaction between H471 and *Xoo*.

### Diverse rice proteins related to signal transduction and transcriptional regulation are involved in the plant-pathogen interaction

During plant-pathogen interactions, plants not only perceive and prevent the pathogen from invading, they also initiate defense responses by activating multi-component systems via modulated expression or abundance and/or post-translational modifications of the associated proteins. After the inoculation with *Xoo*, the accumulation of diverse categories of rice protein kinases and TFs differed significantly between the incompatible and compatible interactions.

Receptor-like kinases (RLKs) reportedly regulate defense processes [28]. On the basis of amino acid sequence and structural differences, RLKs have been categorized into several subfamilies, including leucine-rich repeat RLKs (LRR-RLKs), cysteine-rich repeat RLKs (CRKs), domain of unknown function 26 RLKs, and S-domain RLKs, among others [29]. As common key signaling molecules, CRKs influence diverse stress responses. For example, *OsCRK5* expression is up-regulated in rice in response to the blast fungus or brown planthopper [30, 31]. In the current study, OsCRK5 was more abundant in H471 than in HHZ, in contrast to another CRK, LOC_Os04g25060/LOC_Os04g25650, which was less abundant in H471 than in HHZ. Genes encoding CDPKs are critical for abiotic stress responses [32, 33], but they are also expressed in response to biotic stresses in plants [34]. A previous study indicated that *OsCDPK7* (*OsCDPK13*) expression can be induced by an exposure to chilling stress and high salt concentrations [35]. In the present study, OsCDPK7/OsCDPK13 (LOC_Os04g49510) was significantly more abundant in H471 than in HHZ. The MAPK cascade has been implicated in signaling during plant defense responses to pathogens and various environmental stimuli [36]. In rice, chitin elicitors may activate OsMPK1, OsMPK5, OsMKK4, and the OsMKK4-OsMPK6 cascade to regulate defense activities, including antimicrobial compound biosynthesis, and induce plant cell death [37, 38]. In the current study, OsMKK4 and OsMPK6 accumulated much more in H471 than in HHZ at 1.5–3 dpi. Moreover, 14 phosphatase DAPs, including PP2C30 and two Ser/Thr protein phosphatases, accumulated differently in H471 and HHZ. A previous investigation revealed that PP2C30 interacts with the ABA receptor PYL/RCAR5 and SAPK2 to regulate ABA-dependent gene expression [39]. It also functions as an upstream regulator of *HOX12* expression, acting directly through EUI1 to control panicle exsertion in rice [40].

The bZIP TF family participates in plant responses to abiotic stress via the ABA signaling pathway. For example, *OsbZIP71* RNAi knockdown transgenic lines are highly sensitive to salt, PEG-induced osmotic stress, and ABA [41]. Additionally, OsbZIP23 is a central regulator of ABA signaling and biosynthesis as well as drought tolerance in rice [42]. Moreover, OsbZIP46 positively regulates ABA signaling and drought tolerance in rice, depending on whether it is activated [43]. Our data indicated that two bZIP TFs, namely OsbZIP23 (LOC_Os02g52780) and LOC_Os03g13614, accumulated much more in H471 than in HHZ at 2 and 3 dpi. Furthermore, western blot assays confirmed that OsbZIP23 accumulated more in H471 than in HHZ. These results imply that bZIP TFs are important for the incompatible interaction between rice and *Xoo*.

### Phytoalexin and SA biosynthetic pathways were more enhanced in rice during the incompatible interaction than during the compatible interaction following the *Xoo* infection

In response to pathogens, plants may produce many phytoalexins, including the flavonoid sakuranetin [5]. Sakuranetin is the only known phenolic phytoalexin in rice, and phenylamides are involved in its biosynthesis. The accumulating phenylamides or sakuranetin in plants help reinforce the cell wall and are toxic to pathogens at the infection site [6, 44–46]. Additionally, SA is a small phenolic compound that has an important regulatory role in multiple physiological processes. There are two SA biosynthetic pathways in plants: one involves cinnamate and includes a reaction catalyzed by PAL, whereas the other requires chorismate and includes a reaction catalyzed by ICS [47]. The accumulation of SA always coincides with the up-regulated expression of antimicrobial *PR* genes, which enhances disease resistance responses [7]. Methylated derivatives of SA spread from the infected tissue to distal tissues, thereby inducing systemic acquired resistance [48].

**Fig. 6:**
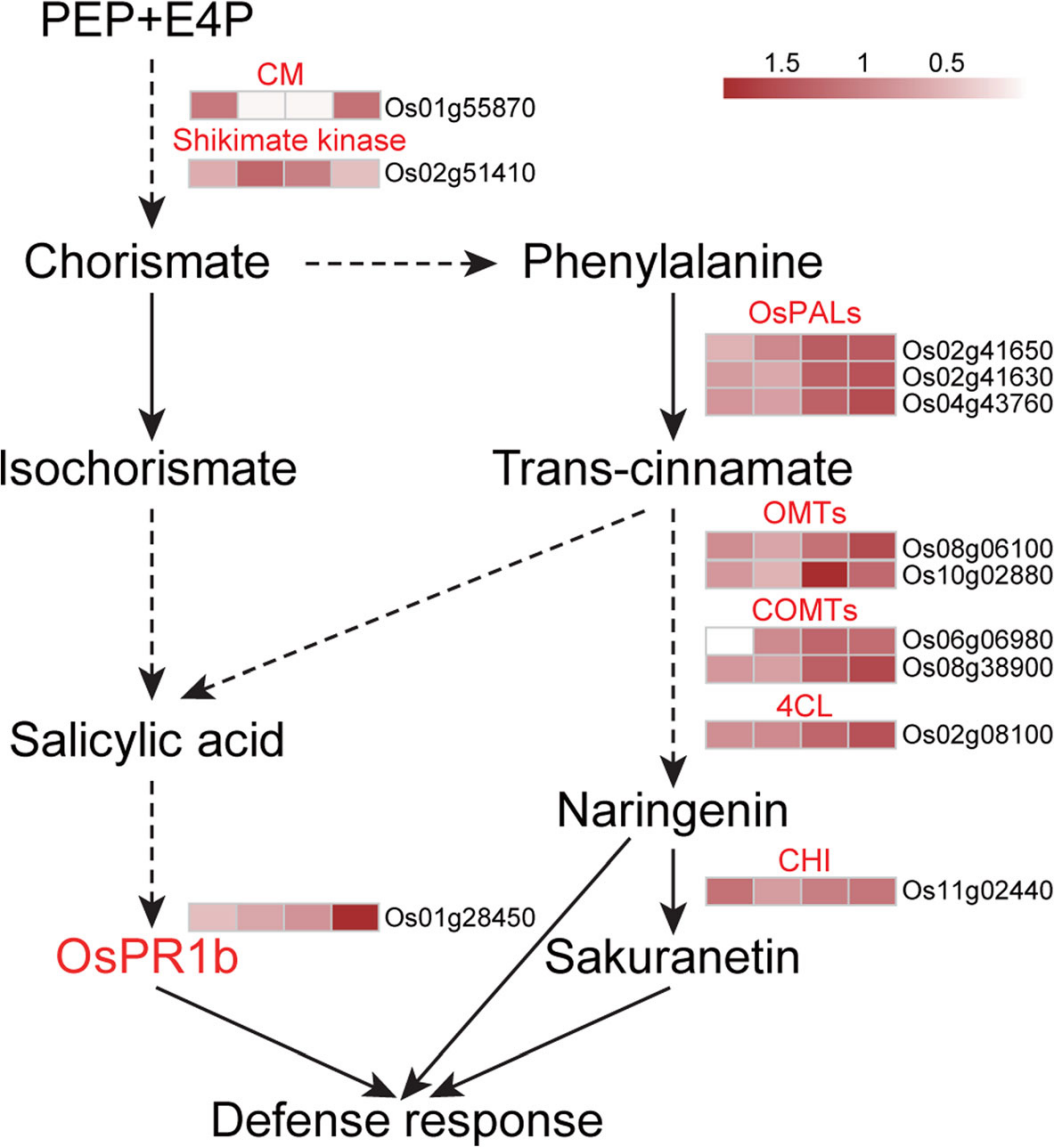
Putative defense pathway underlying the incompatible interaction between H471 and PXO99^A^. Red indicates the proteins were more abundant in H471 than in HHZ. White indicates the proteins were undetectable in this dataset. PEP: phosphoenolpyruvate; E4P: erythrose 4-phosphate; CM: chorismate mutase; ICS: isochorismate synthase; PAL: phenylalanine ammonia-lyase; 4CL: 4-coumarate CoA ligase; COMT: caffeoyl-CoA O-methyltransferase; OMT: O-methyltransferase; CHI: chalcone-flavonone isomerase. Scaled abundance values are presented in the heat map legend. Each row in the heat map corresponds to one protein. The four boxes in each row (left to right) correspond respectively to protein abundances after the inoculation with PXO99^A^ in HHZ at 2 and 3 dpi and in H471 at 2 and 3 dpi

Phenylalanine ammonia-lyases are key enzymes in the phenylpropanoid pathway, which mediates the biosynthesis of the flavonoid-type phytoalexin, sakuranetin, and SA [48]. In the current study, 14 DAPs associated with phenolic phytoalexin biosynthesis were detected. Three PALs (OsPALs) accumulated much more in H471 than in HHZ, whereas no proteins contributing to the chorismite-based SA biosynthetic pathway were identified as a DAP. Furthermore, the SA content increased rapidly in H471 after the inoculation with PXO99^A^, and the accumulation of OsPR1b was about 2-fold higher in H471 than in HHZ. These results suggest that the phytoalexin and SA biosynthetic pathways were activated faster during the incompatible interaction than during the compatible interaction, implying that phytoalexins and SA are involved in the HR of H471 (Fig. 6).

## Conclusion

The results of this study confirm that analyzing proteome-level differences is useful for characterizing plant host-pathogen interactions. The proteomic data presented herein provide a global overview of the protein abundance changes in rice and *Xoo* during their interactions. Further clarifying the functions of DAPs related to *Xoo* virulence and host responses will lead to a more comprehensive understanding of the mechanisms underlying plant host-pathogen interactions.

## Methods

### Plant materials and artificial inoculation

A rice introgression line (IL) (H471) with broad-spectrum BB resistance was selected from a BC_1_F_6_ population derived from a cross between the recurrent parent HHZ and the donor parent PSBRC28. Lines H471 and HHZ were used in this study [16]. Seeds were sown and the germinated seedlings were grown in a seedling nursery with a 13-h light (30 °C): 11-h dark (26 °C) photoperiod. The 30-day-old seedlings were transplanted to a screenhouse at the Institute of Crop Sciences, Chinese Academy of Agricultural Sciences, Beijing, China. Lines H471 and HHZ were artificially inoculated at the early tillering stage with a bacterial solution comprising 10^5^ cells L^**−** 1^ *Xoo* strain PXO99^A^ (Philippines race 6) according to the scissors-dip method [16]. Lesion lengths and the growth curves of PXO99^A^ in H471 and HHZ were evaluated as previously described [16].

### Transmission electron microscopy

The leaves of H471 and HHZ plants inoculated with *Xoo* strain PXO99^A^ were collected at 0 and 3 dpi for the TEM analysis. A 2-mm leaf fragment 1 mm below the lesions was fixed in 2% glutaraldehyde in 0.1 mol L^− 1^ phosphate buffer (pH 7.2) for 4 h at 4 °C and then washed three times in the same buffer. As a post-fixation treatment, the samples were immersed in a 1% osmium tetroxide (OsO_4_) solution for 2 h at room temperature. They were then rinsed three times in the same solution. After staining with 2.5% uranyl acetate, the specimens were dehydrated in a graded ethanol series and then embedded in Spurr’s medium. The samples were polymerized at 40 °C for 24 h and then at 60 °C for 24 h, after which the specimens were cut to produce ultrathin sections (40–60 nm thickness) using the PowerTome-XL ultramicrotome (RMC, AZ, USA). The prepared sections were double-stained with 2% (w/v) uranyl acetate and 2.6% (w/v) lead citrate aqueous solution and then examined with the H-7500 transmission electron microscope (Hitachi Company, Japan).

### Preparation of plant and pathogen samples for the proteomic analysis

In this study, 10 plants per rice line were included in the proteomic analysis. Specifically, 5–10 of the uppermost leaves of each plant were inoculated with PXO99^A^. At 2- and 3-dpi, leaf tips (3 cm long) were collected from three plants (more than three tips per plant) and combined to form one biological replicate, with three independent biological replicates prepared for each genotype at each time-point. The H471 and HHZ leaf samples collected at 2 dpi were designated as H471-2d and HHZ-2d, respectively, whereas the leaf samples collected at 3 dpi were named H471-3d and HHZ-3d, respectively. The reference sample consisted of a mixture of an equal amount of the 12 samples (HHZ-2d-1/2/3, HHZ-3d-1/2/3, H471-2d-1/2/3, and H471-3d-1/2/3).

### Protein extraction and digestion

Tissues were ground in liquid nitrogen and suspended in pre-cooled acetone (− 20 °C) containing 10% (w/v) trichloroacetic acid. After vigorously mixing the solutions, the proteins were precipitated for 16 h at − 20 °C and then collected by centrifuging at 16,000×g (Beckman Allegra 22R) for 30 min at 4 °C. The pelleted proteins were washed three times with pre-cooled acetone. After a lyophilization, the pellets were dissolved in extraction buffer [8 M urea, 50 mM triethylammonium bicarbonate (TEAB), pH 8.5, 1% SDS, and protease inhibitor cocktail (Roche, Indianapolis, USA)]. The soluble protein extracts were collected by centrifuging at 16,000×g for 20 min at 4 °C, after which the protein concentrations were determined with the BCA assay kit (Pierce, Rockford, IL, USA).

### Sample processing for the LC-MS analysis

Tandem mass tags with different reporter ions (126–131 Da) were conjugated to proteins as isobaric tags with the TMT10plex kit (Pierce) for a subsequent relative quantification. Briefly, 100 mM TEAB was added to the protein solution (100 μg total protein) for a final volume of 100 μL. After adding 5 μL 200 mM Tris (2-carboxyethyl) phosphine (TCEP), the mixture was incubated at 55 °C for 1 h. Immediately before use, one tube of iodoacetamide (9 mg) was dissolved in 132 μL 100 mM TEAB to prepare the 375 mM iodoacetamide solution. A 5-μL aliquot of 375 mM iodoacetamide was added to the protein sample and the mixture was incubated for 30 min at room temperature in darkness. Proteins were precipitated with six volumes of pre-chilled (− 20 °C) acetone. The solution was then centrifuged at 8000×g for 10 min at 4 °C and the resulting pellets were washed twice, once with pre-chilled (− 20 °C) 90% (v/v) methanol and then with pre-chilled (− 20 °C) 100% methanol. Protein pellets were briefly dried and resuspended in an adequate amount of 100 mM TEAB (pH 8.5) containing 5% acetonitrile for a concentration of 1 μg/μL. Proteins were digested overnight at 37 °C with 2.5 μg trypsin (Sigma, USA) and then stored at − 80 °C until analyzed. One tube of TMT Label Reagent (Pierce) was added to each 100 μg sample and the labeling reaction was completed at room temperature for 1 h. The reaction was quenched by adding 8 μL 5% hydroxylamine. The samples were labeled with TMT10plex tags. The reference sample was labeled with TMT^10^–131 (Additional file 11). The 12 labeled samples (HHZ-2d-1/2/3, HHZ-3d-1/2/3, H471-2d-1/2/3, and H471-3d-1/2/3) were divided into pools A2 (HHZ-3d-1/2/3 and H471-2d-1/2/3) and B2 (HHZ-2d-1/2/3 and H471-3d-1/2/3). Pools A2 and B2 (30 μg protein per sample) were separately combined with 30 μg labeled reference sample protein in new microcentrifuge tubes.

The two pooled protein samples were lyophilized, reconstituted in solvent A (2% acetonitrile, pH 10), loaded onto an XBridge PST C18 column (130 Å, 5 μm, 250 × 4.6 mm) (Waters, USA), resolved by basic reversed-phase liquid chromatography (off-line) with a gradient of 5–95% solvent B (90% acetonitrile, pH 10) over 40 min, and divided into 20 fractions per pool. The 40 fractions of both pools were collected according to the chromatogram map, concatenated to 20 fractions, centrifuged under a vacuum, and stored at − 80 °C until analyzed by LC-MS/MS.

### LC-MS/MS analysis

The LC-MS/MS analysis involving a Q Exactive hybrid quadrupole orbitrap mass spectrometer (Thermo Scientific, CA, USA) was performed according to the manufacturer-recommended protocols by CapitalBio Technology (China). Briefly, the peptide mixture was separated by reversed-phase chromatography with a DIONEX nano-UPLC system comprising an Acclaim C18 PepMap100 nano-Trap column (75 μm × 2 cm, 2 μm particle size) (Thermo Scientific) connected to an Acclaim PepMap RSLC C18 analytical column (75 μm × 25 cm, 2 μm particle size) (Thermo Scientific). Before loading, samples were dissolved in sample buffer containing 4% acetonitrile and 0.1% formic acid. A linear gradient of mobile phase B (0.1% formic acid in 99.9% acetonitrile) from 3 to 30% in 43 min followed by an increase to 80% mobile phase B in 1 min was applied at a flow rate of 300 nL min^− 1^. The nano-LC was coupled online with the Q Exactive mass spectrometer using a stainless-steel Emitter linked to a nanospray ion source.

The MS survey scan was performed with full scans [350–1600 mass-to-charge ratio (m/z)] acquired with an Orbitrap mass analyzer. The mass resolution was 70,000 at 400 m/z. The 20 most intense precursor ions from a survey scan were selected for MS/MS for each duty cycle and detected at a mass resolution of 35,000 at 400 m/z. All tandem mass spectra were produced by higher-energy collision dissociation. The dynamic exclusion was set for 20 s.

### Protein identification, quantification, and analysis

Proteome Discoverer (version 1.4) software (Thermo Scientific) was used to search the *Oryza sativa* Nipponbare reference genome RGAP 7.0 database [49] and the *Xanthomonas oryzae* pv. *oryzae* PXO99^A^ RefSeq database [50] (32,431 total proteins) with the Sequest algorithms. The precursor mass tolerance and fragment mass tolerance were set to 20 ppm and 0.02 Da, respectively. With the exception of TMT modifications (N-terminus and lysine residues), carbamidomethylation of cysteine was set as a fixed modification and oxidation at methionine was set as a dynamic modification. The digesting enzyme was trypsin, with two missed cleavages allowed. Proteins were quantified with the TMT10plex Isobaric Label Reagent Set (Pierce). Specifically, the unique peptides were used to quantify proteins, with 0 or missing values replaced with the minimum intensity. Additionally, values were rejected if any quantification channels were missing. The false discovery rates (FDRs) of the identified proteins were estimated with the target-decoy approach of Proteome Discoverer. A cutoff value of 1% was used to identify proteins. The protein normalization was conducted as previously described [51]. All protein ratios were normalized against the median protein ratio, which was 1 after the data normalization. The differentially abundant proteins (DAPs) between two samples were identified following a comparison of the mean abundances of three replicates with Student’s *t*-test. The DAPs between H471 and HHZ at each time-point after the PXO99^A^ inoculation (i.e., H471-2d vs HHZ-2d and H471-3d vs HHZ-3d) were screened based on a 1.5-fold change in mean abundance and *p* < 0.05 [52, 53]. All raw mass spectrometry data and search results have been deposited in the iProX database (https://www.iprox.org/), with the data set identifier IPX0001741000 [54].

### Functional analyses based on gene ontology and pathway enrichment

Proteins were functionally analyzed with Gene Ontology (GO) annotations with KOBAS 2.0 [55]. Specifically, the proteins were classified according to the molecular function, biological process, and cellular component categories. The Kyoto Encyclopedia of Genes and Genomes (KEGG) database was screened to identify the enriched pathways among the proteins [56].

For the hierarchical clustering based on different protein functional classifications (GO terms and KEGG pathways), the cluster membership was visualized as a heat map, which was prepared with the heatmap.2 function of the gplots R package.

### Protein immunoblot analysis

Proteins were extracted from HHZ and H471 seedlings with the Plant Protein Extraction Kit (CWBIO, Beijing, China) and quantified with the BSA Protein Assay Kit (CWBIO). Proteins were separated by 10% polyacrylamide gel electrophoresis with protein markers. Polyclonal anti-OsbZIP23, anti-OsCDPK13, anti-OsMKK4, anti-OsMPK6, anti-OsPR1a, and anti-OsPR1b (Beijing Protein Innovation, Beijing, China) were used as primary antibodies (1:2000 dilution), whereas horseradish peroxidase-conjugated goat anti-rabbit (Abmart, Shanghai, China) served as the secondary antibody (1:10,000 dilution). Coomassie Brilliant Blue staining of the large subunit of Rubisco was used as a loading control. The western blot experiment was independently repeated at least three times, with essentially the same results. Representative results are presented.

### Quantification of endogenous salicylic acid

Leaf tips (3 cm long) were collected from HHZ and H471 plants at 0, 1.5, 2.5, 3.5, and 4.5 days after the inoculation with PXO99^A^ and then stored at − 80 °C. The endogenous SA content was quantified as previously described [57]. For each time-point, two biological replicates were analyzed.

## Supplementary Information

**Additional file 1.** Identified rice proteins in two genotypes at two time-points after the inoculation with PXO99^A^.

**Additional file 2 ***Xanthomonas oryzae* pv. *oryzae* (*Xoo*) proteins detected in two genotypes at two time-points after the inoculation with PXO99^A^.

**Additional file 3.** Information regarding all identified peptides.

**Additional file 4.** Information regarding all identified proteins.

**Additional file 5 **Hierarchical cluster analysis of all identified proteins. Clustering of expressed *Xoo* (**a**) and rice (**b**) proteins detected in H471 and HHZ at 2 and 3 days after the inoculation with PXO99^A^ (three biological replicates). Each horizontal line represents a single protein and the colored line indicates the protein abundance relative to the median in a specific sample: red and green indicate high and low accumulations, respectively. H471, rice introgression line with a broad-spectrum hypersensitive reaction mediated by the new rice resistance gene *Xa39*; HHZ, Huang-Hua-Zhan, the recurrent parent of H471.

**Additional file 6 **Differentially abundant *Xanthomonas oryzae* pv. *oryzae* (*Xoo*) proteins between H471 and HHZ at two time-points after the inoculation with PXO99^A^.

**Additional file 7.** Differentially abundant rice proteins between H471 and HHZ at two time-points after the inoculation with PXO99^A^.

**Additional file 8 **GO enrichment analysis of the differentially abundant *Xanthomonas oryzae* pv. *oryzae* (*Xoo*) and rice proteins between H471 and HHZ.

**Additional file 9 **KEGG pathway enrichment analysis of the differentially abundant *Xanthomonas oryzae* pv. *oryzae* (*Xoo*) and rice proteins between H471 and HHZ.

**Additional file 10 **Accumulations of OsPR1a and OsPR1b proteins in HHZ and H471 at different time-points after the inoculation with PXO99^A^. **a** OsPR1a. **b** OsPR1b.

**Additional file 11.** General workflow for the analysis involving isobaric tandem mass tag labeling. Three independent biological replicates of leaf samples were collected for each genotype (H471 and HHZ) at 2 and 3 days after the inoculation with PXO99^A^. Following the protein extraction and quantification of 12 samples (HHZ-2d-1/2/3, HHZ-3d-1/2/3, H471-2d-1/2/3, and H471-3d-1/2/3), an equal amount of each sample was mixed to produce the reference sample. The 13 samples were prepared, digested, and labeled with TMT10plex tags in parallel. The 12 labeled samples (HHZ-2d-1/2/3, HHZ-3d-1/2/3, H471-2d-1/2/3, and H471-3d-1/2/3) were divided into pools A2 (HHZ-3d-1/2/3 and H471-2d-1/2/3) and B2 (HHZ-2d-1/2/3 and H471-3d-1/2/3). Pools A2 and B2 (30 μg protein per sample) were separately combined with 30 μg labeled reference sample protein in new microcentrifuge tubes. The two pooled protein samples were first lyophilized, reconstituted, and fractionated. The peptide mixtures were then analyzed by LC-MS/MS.

**Additional file 12.** All original and uncropped blot images for Fig. 3 and Additional file 10. The images on the left present the OsbZIP23, OsCDPK13, OsMKK4, OsMPK6, PR1a, and PR1b protein abundances in HHZ at 0, 0.5, 1, 1.5, 2, 2.5, 3.5, 4, and 4.5 days after the inoculation with PXO99^A^. The images on the right present the OsbZIP23, OsCDPK13, OsMKK4, OsMPK6, PR1a, and PR1b protein abundances in H471 at 0, 0.5, 1, 1.5, 2, 2.5, 3.5, 4, and 4.5 days after the inoculation with PXO99^A^. The proteins are indicated by arrows.

## Data Availability

All raw mass spectrometry data and search results have been deposited at the iProX (https://www.iprox.org/) with the data set identifiers IPX0001741000.

## Abbreviations

*Xoo*: *Xanthomonas oryzae* pv. *oryzae*
HR: Hypersensitive response
DAPs: Differentially abundant proteins
TMT: Tandem mass tag
LC-MS: Liquid chromatography-quadrupole mass spectrometry
TAL: Transcription activator-like
Omps: Outer membrane proteins
SA: Salicylic acid
NBS-LRR: Nucleotide-binding domain leucine rich repeat
NB-ARC: Nucleotide-binding adaptor shared by APAF-1, R proteins and CED-4

## References

[1] Nino-Liu DO, Ronald PC, Bogdanove AJ. *Xanthomonas oryzae* pathovars: model pathogens of a model crop. Mol Plant Pathol. 2006;7(5):303–24. 10.1111/j.1364-3703.2006.00344.x 20507449

[2] Zhang F, Du Z, Huang L, Vera Cruz C, Zhou Y, Li Z. Comparative transcriptome profiling reveals different expression patterns in *Xanthomonas oryzae* pv. *oryzae* strains with putative virulence-relevant genes. PLoS One. 2013;8(5):e64267. 10.1371/journal.pone.0064267 PMC3667120 23734193

[3] Kanyuka K, Rudd JJ (2019). Cell surface immune receptors: the guardians of the plant's extracellular spaces. Curr Opin Plant Biol.

[4] Liu WD, Liu JL, Triplett L, Leach JE, Wang GL (2014). Novel insights into Rice innate immunity against bacterial and fungal pathogens. Annu Rev Phytopathol.

[5] Ahuja I, Kissen R, Bones AM (2012). Phytoalexins in defense against pathogens. Trends Plant Sci.

[6] Park HL, Yoo Y, Hahn TR, Bhoo SH, Lee SW, Cho MH (2014). Antimicrobial activity of UV-induced phenylamides from rice leaves. Molecules.

[7] Vlot AC, Dempsey DA, Klessig DF (2009). Salicylic acid, a multifaceted hormone to combat disease. Annu Rev Phytopathol.

[8] De Vleesschauwer D, Xu J, Hofte M (2014). Making sense of hormone-mediated defense networking: from rice to Arabidopsis. Front Plant Sci.

[9] Dempsey DA, Vlot AC, Wildermuth MC, Klessig DF (2011). Salicylic acid biosynthesis and metabolism. Arabidopsis Book.

[10] Wildermuth MC, Dewdney J, Wu G, Ausubel FM (2001). Isochorismate synthase is required to synthesize salicylic acid for plant defence. Nature.

[11] Dempsey DA, Klessig DF (2017). How does the multifaceted plant hormone salicylic acid combat disease in plants and are similar mechanisms utilized in humans?. BMC Biol.

[12] Klessig DF, Choi HW, Dempsey DA (2018). Systemic acquired resistance and salicylic acid: past, present, and future. Mol Plant Microbe Interact.

[13] Kim SM, Suh JP, Qin Y, Noh TH, Reinke RF, Jena KK. Identification and fine-mapping of a new resistance gene, *Xa40*, conferring resistance to bacterial blight races in rice (*Oryza sativa* L.). Theor Appl Genet. 2015;128(10):1933–43. 10.1007/s00122-015-2557-2 26081948

[14] Hutin M, Sabot F, Ghesquiere A, Koebnik R, Szurek B. A knowledge-based molecular screen uncovers a broad-spectrum *OsSWEET14* resistance allele to bacterial blight from wild rice. Plant J. 2015;84(4):694–703. 10.1111/tpj.13042 26426417

[15] Busungu C, Taura S, Sakagami JI, Ichitani K (2016). Identification and linkage analysis of a new rice bacterial blight resistance gene from XM14, a mutant line from IR24. Breed Sci.

[16] Zhang F, Zhuo DL, Huang LY, Wang WS, Xu JL, Cruz CV, et al. *Xa39*, a novel dominant gene conferring broad-spectrum resistance to *Xanthomonas oryzae* pv. *oryzae* in rice. Plant Pathol. 2015;64(3):568–75.

[17] Mahmood T, Jan A, Kakishima M, Komatsu S (2006). Proteomic analysis of bacterial-blight defense-responsive proteins in rice leaf blades. Proteomics.

[18] Chen F, Yuan YX, Li Q, He ZH (2007). Proteomic analysis of rice plasma membrane reveals proteins involved in early defense response to bacterial blight. Proteomics.

[19] Wang YM, Kim SG, Wu JN, Huh HH, Lee SJ, Rakwal R, et al. Secretome analysis of the rice bacterium *Xanthomonas oryzae* (*Xoo*) using in vitro and in planta systems. Proteomics. 2013;13(12–13):1901–12. 10.1002/pmic.201200454 23512849

[20] Rauniyar N, Yates JR (2014). Isobaric labeling-based relative quantification in shotgun proteomics. J Proteome Res.

[21] Sharabiani MTA, Siermala M, Lehtinen TO, Vihinen M. Dynamic covariation between gene expression and proteome characteristics. Bmc Bioinformatics. 2005;6(1):215. 10.1186/1471-2105-6-215 PMC1236912 16131395

[22] Miller VL, Bliska JB, Falkow S. Nucleotide sequence of the *Yersinia enterocolitica* ail gene and characterization of the ail protein product. J Bacteriol. 1990;172(2):1062–9. 10.1128/jb.172.2.1062-1069.1990 PMC208537 1688838

[23] Tommassen J, Stoorvogel J, van Bussel MJ, van de Klundert JA. Molecular characterization of an *Enterobacter cloacae* outer membrane protein (OmpX). J Bacteriol. 1991;173(1):156–60. 10.1128/jb.173.1.156-160.1991 PMC207169 1987115

[24] Rott PC, Fleites L, Marlow G, Royer M, Gabriel DW. An OmpA family outer membrane protein is required for both disease symptom development and sugarcane stalk colonization by *Xanthomonas albilineans*. Phytopathology. 2009;99(6):S110–1.

[25] Tang JL, Feng JX, Li QQ, Wen HX, Zhou DL, Wilson TJ, et al. Cloning and characterization of the *rpfC* gene of *Xanthomonas oryzae* pv. *oryzae*: involvement in exopolysaccharide production and virulence to rice. Mol Plant Microbe Interact. 1996;9(7):664–6. 10.1094/mpmi-9-0664 8810082

[26] Romer P, Recht S, Strauss T, Elsaesser J, Schornack S, Boch J, et al. Promoter elements of rice susceptibility genes are bound and activated by specific TAL effectors from the bacterial blight pathogen, *Xanthomonas oryzae* pv. *oryzae*. New Phytol. 2010;187(4):1048–57. 10.1111/j.1469-8137.2010.03217.x 20345643

[27] Cheong H, Kim CY, Jeon JS, Lee BM, Moon JS, Hwang I. *Xanthomonas oryzae* pv. *oryzae* type III effector XopN targets OsVOZ2 and a putative thiamine synthase as a virulence factor in Rice. PLoS One. 2013;8(9):e73346. 10.1371/journal.pone.0073346 PMC3760903 24019919

[28] Osakabe Y, Maruyama K, Seki M, Satou M, Shinozaki K, Yamaguchi-Shinozaki K (2005). Leucine-rich repeat receptor-like kinase1 is a key membrane-bound regulator of abscisic acid early signaling in Arabidopsis. Plant Cell.

[29] Shiu SH, Bleecker AB (2001). Receptor-like kinases from Arabidopsis form a monophyletic gene family related to animal receptor kinases. Proc Natl Acad Sci U S A.

[30] Kim ST, Kim SG, Hwang DH, Kang SY, Kim HJ, Lee BH, et al. Proteomic analysis of pathogen-responsive proteins from rice leaves induced by rice blast fungus, *Magnaporthe grisea*. Proteomics. 2004;4(11):3569–78. 10.1002/pmic.200400999 15478215

[31] Wei Z, Hu W, Lin Q, Cheng X, Tong M, Zhu L, et al. Understanding rice plant resistance to the Brown Planthopper (*Nilaparvata lugens*): a proteomic approach. Proteomics. 2009;9(10):2798–808. 10.1002/pmic.200800840 19405033

[32] Ludwig AA, Romeis T, Jones JD (2004). CDPK-mediated signalling pathways: specificity and cross-talk. J Exp Bot.

[33] Saijo Y, Hata S, Kyozuka J, Shimamoto K, Izui K. Over-expression of a single Ca^2+^−dependent protein kinase confers both cold and salt/drought tolerance on rice plants. Plant J. 2000;23(3):319–27. 10.1046/j.1365-313x.2000.00787.x 10929125

[34] Romeis T, Herde M (2014). From local to global: CDPKs in systemic defense signaling upon microbial and herbivore attack. Curr Opin Plant Biol.

[35] Wan B, Lin Y, Mou T. Expression of rice Ca^2+^-dependent protein kinases (CDPKs) genes under different environmental stresses. FEBS Lett. 2007;581(6):1179–89. 10.1016/j.febslet.2007.02.030 17336300

[36] Meng X, Zhang S (2013). MAPK cascades in plant disease resistance signaling. Annu Rev Phytopathol.

[37] Kishi-Kaboshi M, Okada K, Kurimoto L, Murakami S, Umezawa T, Shibuya N (2010). A rice fungal MAMP-responsive MAPK cascade regulates metabolic flow to antimicrobial metabolite synthesis. Plant J.

[38] Yoo SJ, Kim SH, Kim MJ, Ryu CM, Kim YC, Cho BH, et al. Involvement of the OsMKK4-OsMPK1 cascade and its downstream transcription factor OsWRKY53 in the wounding response in Rice. Plant Pathology J. 2014;30(2):168–77. 10.5423/PPJ.OA.10.2013.0106 PMC4174855 25288999

[39] Kim H, Hwang H, Hong JW, Lee YN, Ahn IP, Yoon IS (2012). A rice orthologue of the ABA receptor, OsPYL/RCAR5, is a positive regulator of the ABA signal transduction pathway in seed germination and early seedling growth. J Exp Bot.

[40] Gao SP, Fang J, Xu F, Wang W, Chu CC. Rice HOX12 regulates panicle Exsertion by directly modulating the expression of *ELONGATED UPPERMOST INTERNODE1*. Plant Cell. 2016;28(3):680–95. 10.1105/tpc.15.01021 PMC4826014 26977084

[41] Zhang CY, Liu J, Zhao T, Gomez A, Li C, Yu CS (2016). A drought-inducible transcription factor delays reproductive timing in Rice. Plant Physiol.

[42] Xiang Y, Tang N, Du H, Ye HY, Xiong LZ. Characterization of OsbZIP23 as a key player of the basic Leucine zipper transcription factor family for conferring abscisic acid sensitivity and salinity and drought tolerance in Rice. Plant Physiol. 2008;148(4):1938–52. 10.1104/pp.108.128199 PMC2593664 18931143

[43] Tang N, Zhang H, Li XH, Xiao JH, Xiong LZ (2012). Constitutive activation of transcription factor OsbZIP46 improves drought tolerance in Rice. Plant Physiol.

[44] Edreva AM, Velikova VB, Tsonev TD (2007). Phenylamides in plants. Russ J Plant Physl+.

[45] von Roepenack-Lahaye E, Newman MA, Schornack S, Hammond-Kosack KE, Lahaye T, Jones JDG (2003). P-coumaroylnoradrenaline, a novel plant metabolite implicated in tomato defense against pathogens. J Biol Chem.

[46] Yang Q, He Y, Kabahuma M, Chaya T, Kelly A, Borrego E, et al. A gene encoding maize caffeoyl-CoA O-methyltransferase confers quantitative resistance to multiple pathogens. Nat Genet. 2017;49:1364–72. 10.1038/ng.3919 28740263

[47] Yang L, Li BS, Zheng XY, Li JG, Yang M, Dong XN, et al. Salicylic acid biosynthesis is enhanced and contributes to increased biotrophic pathogen resistance in Arabidopsis hybrids. Nat Commun. 2015;6:7309. 10.1038/ncomms8309 PMC4490401 26065719

[48] Duan L, Liu HB, Li XH, Xiao JH, Wang SP. Multiple phytohormones and phytoalexins are involved in disease resistance to *Magnaporthe oryzae* invaded from roots in rice. Physiol Plant. 2014;152(3):486–500. 10.1111/ppl.12192 24684436

[49] Kawahara Y, de la Bastide M, Hamilton JP, Kanamori H, McCombie WR, Ouyang S (2013). Improvement of the *Oryza sativa* Nipponbare reference genome using next generation sequence and optical map data. Rice (N Y).

[50] Salzberg SL, Sommer DD, Schatz MC, Phillippy AM, Rabinowicz PD, Tsuge S, et al. Genome sequence and rapid evolution of the rice pathogen *Xanthomonas oryzae* pv. *oryzae* PXO99^A^. BMC Genomics. 2008;9:204. 10.1186/1471-2164-9-204 PMC2432079 18452608

[51] Ross PL, Huang YN, Marchese JN, Williamson B, Parker K, Hattan S, et al. Multiplexed protein quantitation in *Saccharomyces cerevisiae* using amine-reactive isobaric tagging reagents. Mol Cell Proteomics. 2004;3(12):1154–69. 10.1074/mcp.M400129-MCP200 15385600

[52] Hu J, Ren B, Dong S, Liu P, Zhao B, Zhang J (2020). Comparative proteomic analysis reveals that exogenous 6-benzyladenine (6-BA) improves the defense system activity of waterlogged summer maize. BMC Plant Biol.

[53] Wang X, Shi T, Zhao Z, Hou H, Zhang L. Proteomic analyses of sheep (*Ovis aries*) embryonic skeletal muscle. Sci Rep. 2020;10(1):1750. 10.1038/s41598-020-58349-0 PMC7000794 32019949

[54] Ma J, Chen T, Wu S, Yang C, Bai M, Shu K (2019). iProX: an integrated proteome resource. Nucleic Acids Res.

[55] Xie C, Mao X, Huang J, Ding Y, Wu J, Dong S (2011). KOBAS 2.0: a web server for annotation and identification of enriched pathways and diseases. Nucleic Acids Res.

[56] Kanehisa M, Goto S (2000). KEGG: Kyoto encyclopedia of genes and genomes. Nucleic Acids Res.

[57] Chen ML, Fu XM, Liu JQ, Ye TT, Hou SY, Huang YQ (2012). Highly sensitive and quantitative profiling of acidic phytohormones using derivatization approach coupled with nano-LC-ESI-Q-TOF-MS analysis. J Chromatogr B.

