# Supplementary figures and images for: Comparative proteomic analysis reveals novel insights into the interaction between rice and Xanthomonas oryzae pv. oryzae
Source: BMC Plant Biol. 2020 Dec 14;20:563. doi: 10.1186/s12870-020-02769-7 (PMC7734852; doi:10.1186/s12870-020-02769-7)

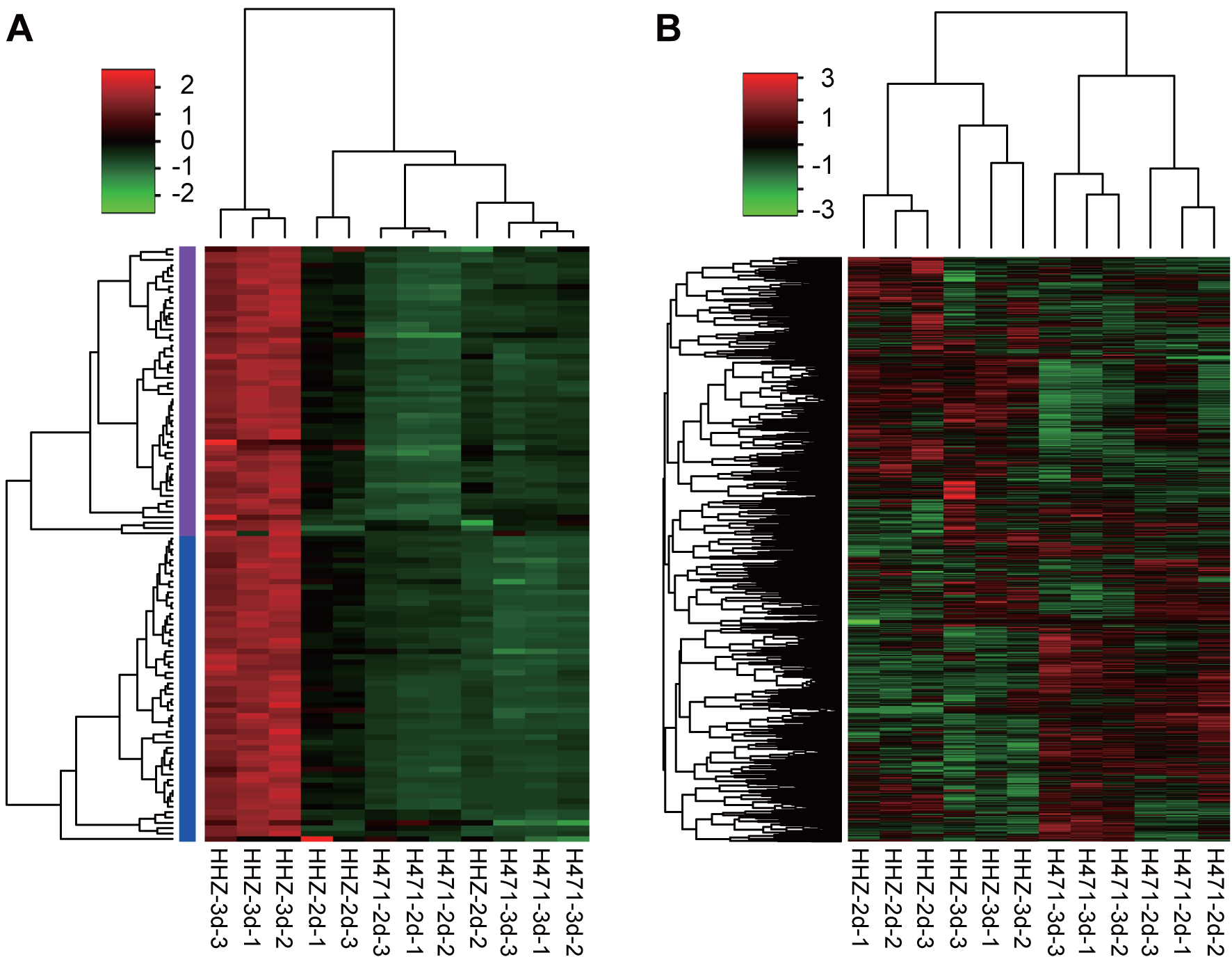

Supplement: Supplementary file 5 — Additional file 5 Hierarchical cluster analysis of all identified proteins. Clustering of expressed Xoo (a) and rice (b) proteins detected in H471 and HHZ at 2 and 3 days after the inoculation with PXO99A (three biological replicates). Each horizontal line represents a single protein and the colored line indicates the protein abundance relative to the median in a specific sample: red and green indicate high and low accumulations, respectively. H471, rice introgression line with a broad-spectrum hypersensitive reaction mediated by the new rice resistance gene Xa39; HHZ, Huang-Hua-Zhan, the recurrent parent of H471. [file 12870_2020_2769_MOESM5_ESM.tif]

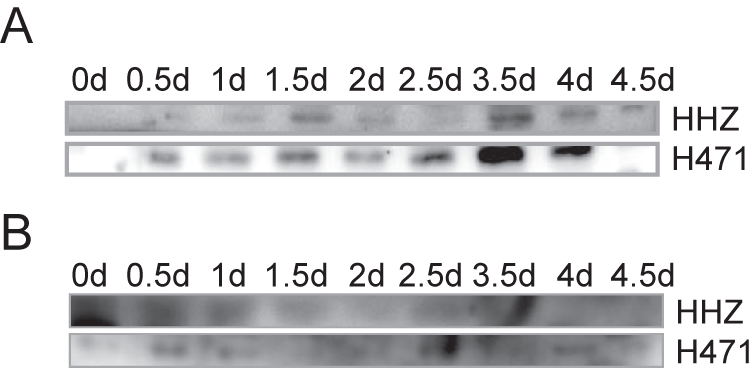

Supplement: Supplementary file 10 — Additional file 10 Accumulations of OsPR1a and OsPR1b proteins in HHZ and H471 at different time-points after the inoculation with PXO99A. a OsPR1a. b OsPR1b. [file 12870_2020_2769_MOESM10_ESM.tif]

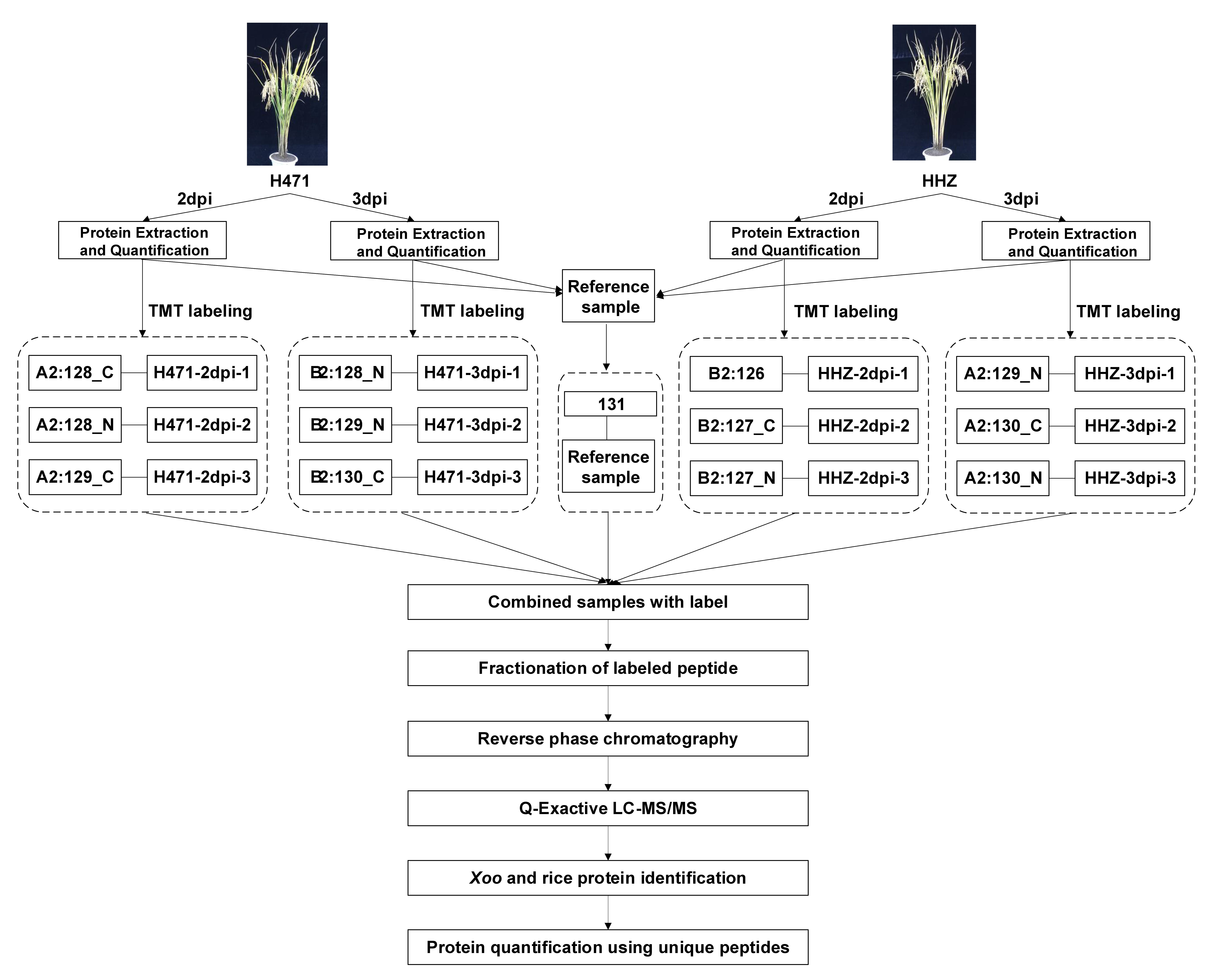

Supplement: Supplementary file 11 — Additional file 11. General workflow for the analysis involving isobaric tandem mass tag labeling. Three independent biological replicates of leaf samples were collected for each genotype (H471 and HHZ) at 2 and 3 days after the inoculation with PXO99A. Following the protein extraction and quantification of 12 samples (HHZ-2d-1/2/3, HHZ-3d-1/2/3, H471-2d-1/2/3, and H471-3d-1/2/3), an equal amount of each sample was mixed to produce the reference sample. The 13 samples were prepared, digested, and labeled with TMT10plex tags in parallel. The 12 labeled samples (HHZ-2d-1/2/3, HHZ-3d-1/2/3, H471-2d-1/2/3, and H471-3d-1/2/3) were divided into pools A2 (HHZ-3d-1/2/3 and H471-2d-1/2/3) and B2 (HHZ-2d-1/2/3 and H471-3d-1/2/3). Pools A2 and B2 (30 μg protein per sample) were separately combined with 30 μg labeled reference sample protein in new microcentrifuge tubes. The two pooled protein samples were first lyophilized, reconstituted, and fractionated. The peptide mixtures were then analyzed by LC-MS/MS. [file 12870_2020_2769_MOESM11_ESM.tif]
