# Supplementary material for: Comparative proteomic analysis reveals novel insights into the interaction between rice and Xanthomonas oryzae pv. oryzae
Source: BMC Plant Biol. 2020 Dec 14;20:563. doi: 10.1186/s12870-020-02769-7 (PMC7734852; doi:10.1186/s12870-020-02769-7)
Supplement: Supplementary file 12 — Additional file 12. All original and uncropped blot images for Fig. 3 and Additional file 10. The images on the left present the OsbZIP23, OsCDPK13, OsMKK4, OsMPK6, PR1a, and PR1b protein abundances in HHZ at 0, 0.5, 1, 1.5, 2, 2.5, 3.5, 4, and 4.5 days after the inoculation with PXO99A. The images on the right present the OsbZIP23, OsCDPK13, OsMKK4, OsMPK6, PR1a, and PR1b protein abundances in H471 at 0, 0.5, 1, 1.5, 2, 2.5, 3.5, 4, and 4.5 days after the inoculation with PXO99A. The proteins are indicated by arrows. [file 12870_2020_2769_MOESM12_ESM.pptx]

## Slide 1
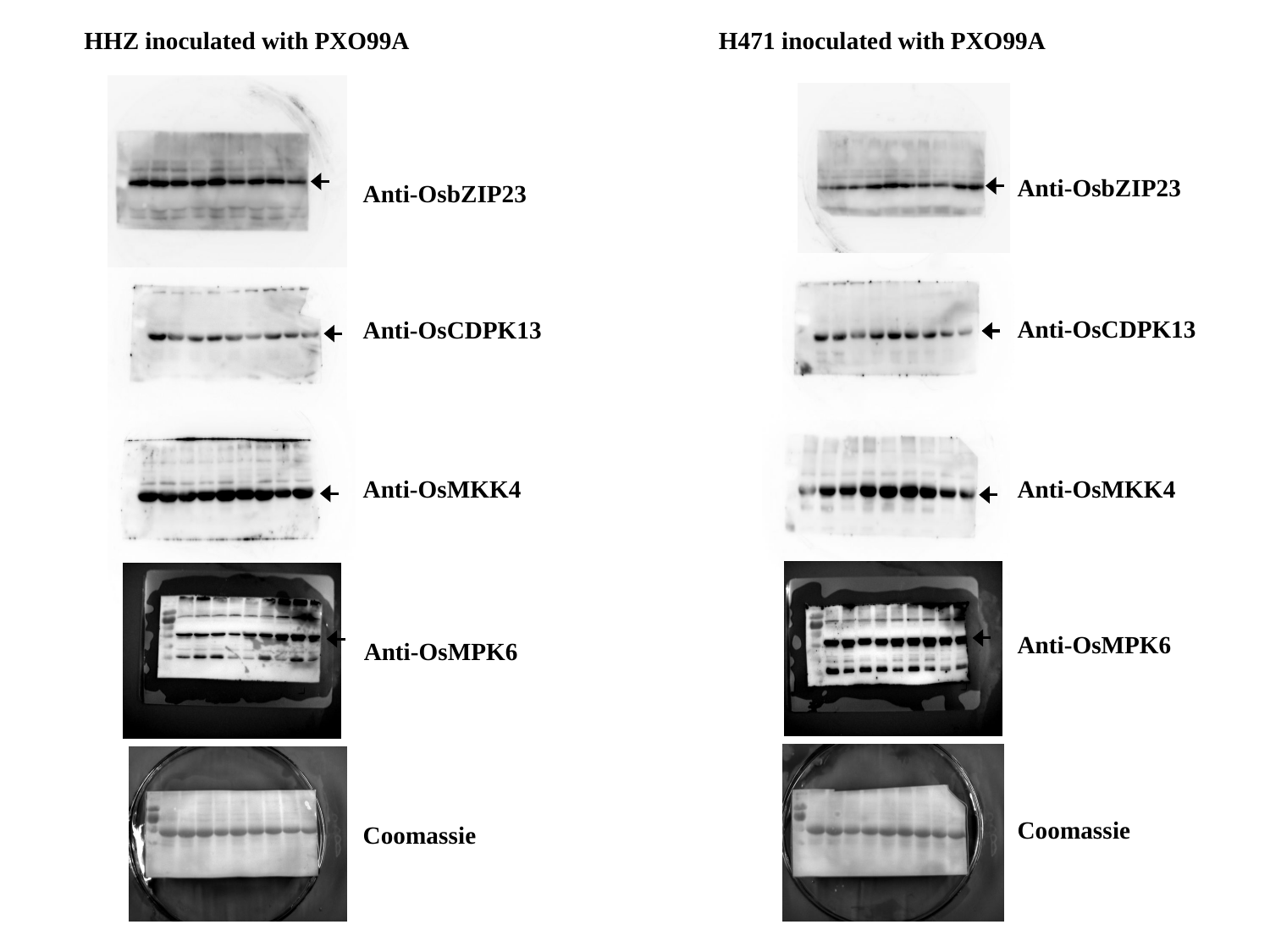

HHZ inoculated with PXO99A
H471 inoculated with PXO99A
Anti-OsbZIP23
Anti-OsbZIP23
Anti-OsCDPK13
Anti-OsCDPK13
Anti-OsMKK4
Anti-OsMKK4
Anti-OsMPK6
Anti-OsMPK6
Coomassie
Coomassie

## Slide 2
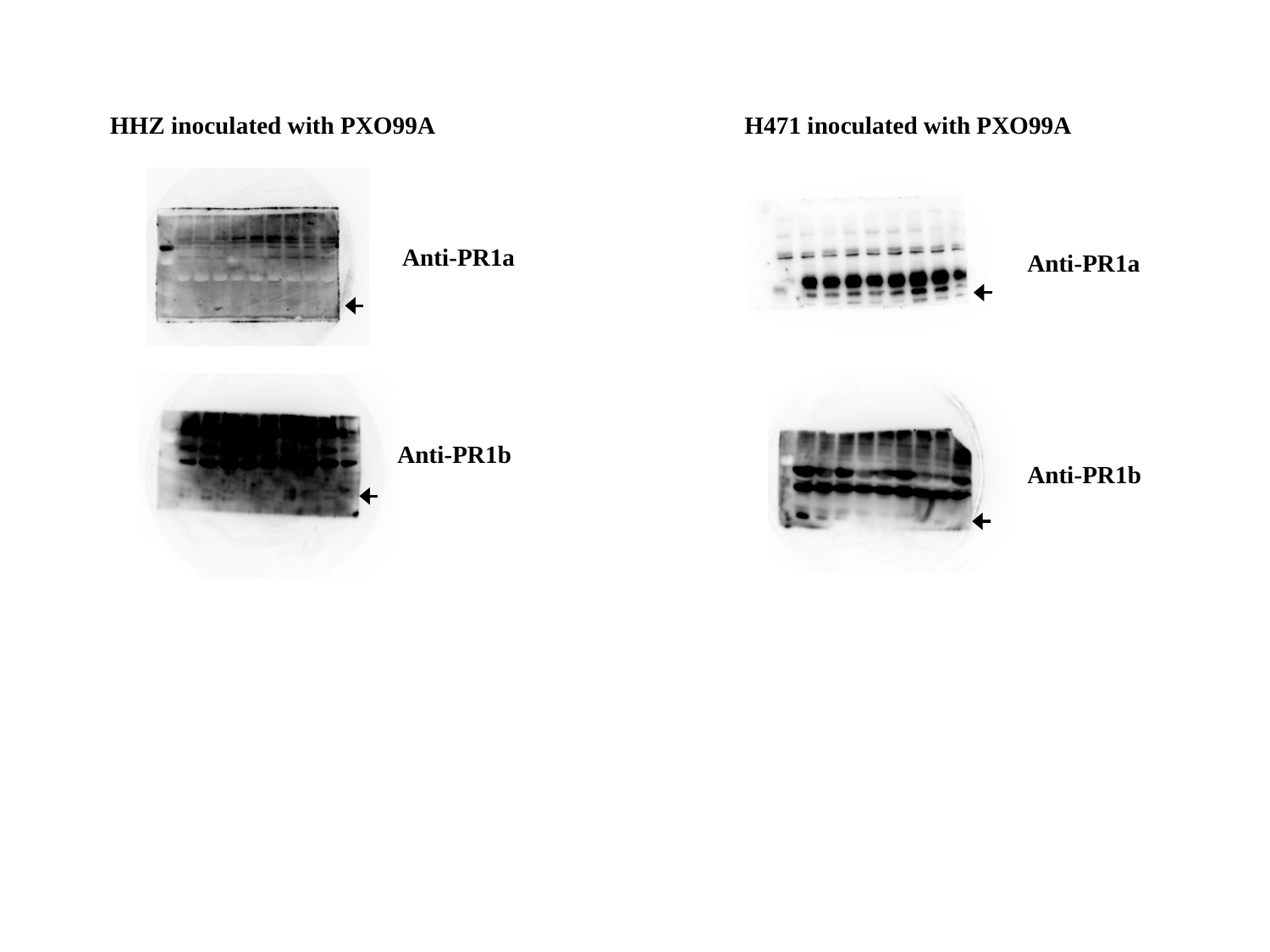

HHZ inoculated with PXO99A
H471 inoculated with PXO99A
Anti-PR1a
Anti-PR1a
Anti-PR1b
Anti-PR1b
